# Níveis mais Baixos de Fetuína-A Sérica estão Associados a um Maior Risco de Mortalidade em Dez Anos em Pacientes com Infarto do Miocárdio por Supradesnivelamento do Segmento ST

**DOI:** 10.36660/abc.20201057

**Published:** 2021-11-17

**Authors:** Hakan Çakır, Selçuk Kanat, Hilal Çakır, Erhan Tenekecioğlu

**Affiliations:** 1 Kartal Kosuyolu Training and Research Hospital Istanbul Turquia Kartal Kosuyolu Training and Research Hospital , Istanbul – Turquia; 2 Bursa Yuksek Ihtisas Training and Research Hospital Bursa Turquia Bursa Yuksek Ihtisas Training and Research Hospital , Bursa – Turquia; 3 Pendik State Hospital Istanbul Turquia Pendik State Hospital , Istanbul – Turquia

**Keywords:** Infarto do Miocárdio com Supradesnível do Segmento ST, Doença da Artéria Coronariana, Mortalidade, Fetuína A, Globulinas, Anti-Inflamatórios, Ecocardiografia/métodos

## Abstract

**Fundamento:**

A fetuína-A é um fator anti-inflamatório e anticalcificação envolvido no curso da doença arterial coronariana (DAC). Em alinhamento com essas funções, investigou-se a fetuína-A como marcador de risco cardiovascular em vários estudos. Porém, a associação entre a fetuína-A e o prognóstico dos pacientes com DAC ainda é controversa.

**Objetivos:**

O presente estudo foi conduzido para identificar a associação entre o nível de fetuína-A sérica e doença cardiovascular (DCV) de longo prazo e a mortalidade global por infarto do agudo do miocárdio por supradesnivelamento do segmento ST (STEMI).

**Métodos:**

Foram cadastrados no estudo cento e oitenta pacientes consecutivos com STEMI. A população do estudo foi dividida em subgrupos (mais baixo, ≤288 µg/ml; e mais alto, >288 µg/ml) de acordo com a mediana do nível de fetuína-A. Dados de acompanhamento clínico foram obtidos por contato telefônico anual com pacientes ou familiares. As causas das mortes também foram confirmadas pelo banco de dados de saúde nacional. P-valores bilaterais <0,05 foram considerados estatisticamente significativos.

**Resultados:**

Durante um acompanhamento médio de 10 anos, foram registradas 71 mortes, das quais 62 foram devidas a DCV. Identificou-se um índice de mortalidade global e por DCV significativamente mais alto no grupo com nível de fetuína-A mais baixo que no grupo com nível de fetuína-A mais alto (44% versus 24%, p= 0,005; 48% versus 31%, p= 0,022, respectivamente). Nas análises de risco proporcionais por regressão de Cox, detectou-se que a fetuína-A era um preditor independente de mortalidade global e por DCV.

**Conclusões:**

A baixa concentração de fetuína-A está associada ao prognóstico de longo prazo ruim pós-STEMI, independentemente de fatores de risco cardiovascular tradicionais. Nossos achados fortaleceram estudos prévios demonstrando consistentemente o papel determinante dos mediadores anti-inflamatórios em síndromes coronárias agudas.

## Introdução

A fetuína-A, glicoproteína alfa-2-Heremans-Schmid, é uma proteína sérica abundantes que é produzida exclusivamente pelo fígado, pela língua, pela placenta e pelo tecido adiposo.^1 , 2^ Ela tem um papel crucial como inibidor fisiológico do receptor tirosina quinase associado à resistência a insulina e um reagente de fase aguda negativa. Ela também regula a remodelação óssea e o metabolismo do cálcio, sendo um inibidor importante da precipitação de sais de cálcio e calcificações vasculares.^3^

A inflamação é um processo-chave na aterosclerose. Existem evidências significativas do efeito prejudicial às citocinas pró-inflamatórias no sistema cardiovascular.^4^ Durante a inflamação, citocinas pró-inflamatórias, tais como IL-1ß e IL-6, reduzem a síntese da fetuína-A no fígado. Níveis de fetuína-A circulante reduzidos limitam as atividades de inúmeros mediadores anti-inflamatórios, agravando, dessa forma, a resposta inflamatória. A fetuína-A é um dos inibidores de calcificação em tecido mole e na árvore vascular.^5^ A calcificação da artéria coronária é vista como índice da gravidade da doença vascular aterosclerótica e pode também prever eventos cardiovasculares adversos futuros.

Em alinhamento com essas funções, investigou-se a fetuína-A como marcador de risco cardiovascular em vários estudos. Estudos em populações com doença renal terminal (DRT) demonstram consistentemente que níveis de fetuína-A mais baixos são associados aos eventos de DCV e mortalidade global.^6 - 8^ Entretanto, a relação entre os níveis de fetuína-A sérica e o prognóstico de pacientes com doença arterial coronariana (DAC) ainda é controversa. Níveis altos de fetuína-A sérica estão associados a uma mortalidade de um ano mais baixa nos casos de síndromes coronárias agudas (SCA).^9^ Por outro lado, Parker et al.,^10^ demonstraram que não se observou associação significativa entre a fetuína-A e evento de mortalidade ou DCV em pacientes com DAC estável.^10^ Weikert et al.,^11^ relataram uma relação entre níveis de fetuína-A plasmática aumentada, e riscos mais altos de infarto do miocárdio (IM) e acidente vascular isquêmico em um estudo coorte de grande população.^11^

No presente estudo, o objetivo foi identificar a associação entre o nível de fetuína-A sérica e DCV de longo prazo e a mortalidade global de infarto do agudo do miocárdio por supradesnivelamento do segmento ST (STEMI). Levantou-se a hipótese de que níveis de fetuína-A mais baixos sejam um marcador prognóstico para mortalidade em longo prazo pós-STEMI, independentemente de outros fatores de risco de DCV.

## Métodos

### População do estudo

Entre maio e setembro de 2009, todos os pacientes (n: 195) diagnosticados com STEMI, e internados em até 12 horas após o surgimento dos sintomas, foram cadastrados inicialmente no estudo. Pacientes com choque cardiogênico em até 24 horas também foram incluídos. O STEMI foi diagnosticado na presença dos dois critérios abaixo: angina persistente por ≥ 20 minutos e supradesnivelamento do segmento ST de ≥ 1 mm em ≥ 2 derivações contíguas além de V2 ou V3, ou a presença de novos bloqueios de ramos do feixe esquerdo. Nas derivações V2 a V3, 2 mm de supradesnivelamento de ST nos homens, e 1,5 mm de supradesnivelamento de ST em mulheres, eram necessários para o diagnóstico de STEMI. Os pacientes foram manejados de acordo com as diretrizes, e o tratamento não foi afetado pela participação no cadastro. Preferiu-se a intervenção coronária percutânea (ICP) primária como técnica de reperfusão para toda a população do estudo. Os pacientes foram excluídos se alguma das características abaixo estivesse presente: lesão culpada ou crítica na artéria coronária principal esquerda, cirurgia de bypass da artéria coronária anterior, doença renal terminal (clearance de creatinina, <15 mL/min), infecção ativa, doença inflamatória crônica, e malignidade conhecida. Ao final, a população do estudo consistiu em 180 pacientes. Foi realizado o exame físico completo e obteve-se um histórico médico detalhado de cada caso. Altura, peso e pressão arterial (PA) foram medidos por enfermeiros treinados, utilizando-se protocolos e procedimentos padrão. Hipertensão foi definida pela pressão arterial sistólica ≥ 140 mmHg, ou pressão arterial diastólica ≥ 90 mmHg, ou tratamento atual com qualquer medicamento hipertensivo. O índice de massa corporal (IMC) foi calculado como peso (kg)/ altura^2^ (m^2^). O termo de consentimento informado foi obtido de todos os participantes. O estudo foi analisado e aprovado pelos comitês de ética institucionais.

### Medidas bioquímicas

Foram coletadas amostras de sangue venoso dos participantes após um jejum noturno. O soro foi separado imediatamente das células por centrifugação a 3000 g por 10 minutos, armazenado a frio a -70 °C até ser analisado. A fetuína-A sérica foi medida, utilizando-se um kit de ensaio de imunoabsorção enzimática de fetuína-A humana disponível comercialmente (BioVendor Laboratory Medicine, Inc., Brno, República Checa). Os coeficientes de variação intra e interensaio da fetuína-A foram menores que 8%. Os níveis ultrassensíveis de PCR (PCR-us) foram medidos pelo método imunonefelométrico (Image Immunochemistry System; Beckman Coulter, Inc., Fullerton, CA, EUA). Outros parâmetros bioquímicos foram medidos utilizando-se métodos e kits comercialmente disponíveis.

### Ecocardiográfica

Foi realizado um ecocardiograma bidimensional no hospital, antes da alta, para avaliar a fração de ejeção do ventrículo esquerdo (FEVE), utilizando-se a técnica de Simpson modificada. A análise foi realizada por dois observadores cegos em relação aos dados clínicos e angiográficos.

### Acompanhamento prospectivo e resultados

Dados de acompanhamento clínico foram obtidos por contato telefônico anual com pacientes ou familiares após o ICP. As causas das mortes também foram confirmadas pelo banco de dados de saúde nacional. O acompanhamento foi concluído para todos os pacientes. Os resultados principais do estudo foram mortalidade cardiovascular (CID I00-I99) e mortalidade global, que incluem mortalidade cardiovascular e morte devido a causas não cardíacas. Os pacientes passaram por triagens até o final de fevereiro de 2019 ou até a morte do paciente.

### Análise estatística

Variáveis contínuas com distribuição normal são expressas como médias ± desvio padrão (DP) e variáveis contínuas sem distribuição normal são expressas como mediana e faixa interquartil (FIQ). Dados categóricos são expressos como valores absolutos e porcentagens. O teste de Kolmogorov-Smirnov foi utilizado para testar a distribuição normal. As diferenças em variáveis contínuas entre os grupos foram analisadas utilizando o teste t de amostras independentes ou o teste U de Mann-Whitney, para variáveis com o sem distribuição normal, respectivamente. Variáveis categóricas e proporções foram analisadas pelo teste Qui-quadrado. A correlação entre a fetuína-A plasmática e os fatores de risco de DCV foi analisada utilizando-se a correlação de Pearson. Curvas de sobrevida para subgrupos de fetuína-A plasmática foram construídas pelo método Kaplan-Meier, e as diferenças em sobrevida foram avaliadas utilizando-se o teste de Log-rank.

A análise do modelo de risco proporcional de Cox foi usada para avaliar a associação entre a fetuína-A sérica e mortalidade global e por DCV no acompanhamento de 10 anos. O primeiro modelo era não padronizado. O modelo 2 foi padronizado para idade e sexo. Com base no modelo 2, o modelo 3 foi padronizado além disso para fatores de risco cardiovascular tradicionais, tais como tabagismo, histórico de diabetes mellitus, histórico de hipertensão, e nível de colesterol total. Com base no modelo 3, o modelo 4 foi padronizado além disso para nível de creatinina e FEVE. A fetuína-A foi analisada separadamente como variável categórica e variável contínua. Os resultados dos modelos de regressão de Cox foram apresentados como razões de chance (RC) e intervalos de confiança (IC) de 95%.

P-valores bilaterais <0,05 foram considerados estatisticamente significativos. As análises estatísticas foram realizadas com o software SPSS 20.0 (SPSS Inc., Chicago, IL, EUA).

## Resultados

### Características clínicas e bioquímicas

A população do estudo era composta de 180 pacientes com STEMI e dividida em subgrupos com fetuína-A mais baixa e mais alta, de acordo com a mediana do nível de fetuína-A na admissão. As características demográficas e bioquímicas dos níveis de fetuína-A na linha de base de acordo com os subgrupos (mais baixo, ≤288 µg/ml; e mais alto, >288 µg/ml) estão resumidas na Tabela 1 . Os pacientes do grupo com fetuína-A mais baixa eram mais velhos, e tinham uma prevalência mais alta de hipertensão e níveis mais altos de creatinina sérica e PCR-us em comparação ao grupo com fetuína-A mais alta. No ecocardiograma, os valores de FEVE eram mais baixos no grupo com fetuína-A mais baixa.

**Tabela 1:** – Características de linha de base da população do estudo estratificada pela mediana dos valores de fetuína-A na admissão (≤288 µg/ml versus ≤288 µg/ml)

Variáveis	Grupos de fetuína-A		p-valor
	Fetuína-A mais baixa (≤288 µg/ml) (n:90)	Fetuína-A mais alta (≤288 µg/ml) (n:90)	
Idade, a	60,5±9,9	57,1±9,6	0,019
Sexo, feminino/masculino	12/78	17/73	0,311
IMC, kg/m^2^	26,0±2,0	25,8±2,0	0,528
Tabagismo, n (%)	59 (65,6)	51 (56,7)	0,221
Hipertensão, n (%)	32 (35,6)	19 (21,1)	0,032
Diabetes mellitus, n (%)	25 (27,8)	18 (20,0)	0,221
Colesterol total, mg/dl	181±34	182±34	0,922
*Triglicérides, mg/dl	141 [102-194]	137 [103-181]	0,595
HDL colesterol, mg/dl	33±6	35±6	0,059
LDL colesterol, mg/dl	115±30	113±31	0,658
Hemoglobina, g/dl	12,7±1,46	13,0±1,18	0,240
Creatinina, mg/dl	1,10±0,74	0,84±0,30	0,002
PCR-us, mg/dl	1,91±2,31	0,86±1,56	<0,001
Pico de troponina I, ng/ml	45,0±37,6	35,1±34,1	0,066
IM prévio, n (%)	6 (6,6)	4 (5,5)	0,756
IM da parede anterior, n (%)	35 (38,9)	28 (31,1)	0,274
IM inferior ou ventr. direito, n (%)	40 (44,4)	54 (60,0)	0,037
IM de parede posterolateral, n (%)	15 (16,7)	8 (8,9)	0,118
Tempo de reperfusão, h	5,7±6,8	6,4±10,0	0,547
Administração de tirofibana, n (%)	55 (61,1)	50 (55,6)	0,450
FEVE, %	42±7	45±6	0,016

Os dados são expressos como média ± DP, n (%) ou *mediana [FIQ]. IMC: índice de massa corporal; HDL: lipoproteína de alta densidade; PCR-us: proteína C reativa ultrassensível; IM: infarto do miocárdio; LDL: lipoproteína de baixa densidade; FEVE: fração de ejeção ventricular esquerda.

### Fetuína-A e fatores de risco de DCV

A análise de correlação bivariada entre fetuína-A sérica e fatores de risco de DCV é apresentada na Tabela 2 . O nível de fetuína-A estava inversamente correlacionado ao PCR-us, e diretamente correlacionado à glicemia de jejum após a padronização por sexo, idade e IMC.

**Tabela 2:** – Associação de fetuína-A plasmática com fatores de risco de doença cardiovascular

	Correlação com os níveis de fetuína-A plasmática			
	Sem padronização		Padronizado para Sexo, idade e IMC	
Variáveis	r	p-valor^†^	R	p-valor^‡^
Idade, a	-0,245	0,001	-	-
IMC, kg/m^2^	0,031	0,681	-	-
PAS, mm Hg	0,084	0,263	0,038	0,616
PAD, mm Hg	0,063	0,403	0,018	0,810
Glicemia jejum, mg/dl	0,288	<0,001	0,272	<0,001
Colesterol total, mg/dl	0,056	0,456	-0,004	0,961
Triglicérides, mg/dl	0,083	0,268	0,073	0,333
HDL colesterol, mg/dl	0,175	0,019	0,124	0,099
LDL colesterol, mg/dl	-0,009	0,903	-0,078	0,302
PCR-us, mg/dl	-0,276	<0,001	-0,233	0,002

*IMC: índice de massa corporal; PAD: pressão arterial diastólica; HDL: lipoproteína de alta densidade; PCR-us: proteína C reativa ultrassensível; LDL: lipoproteína de baixa densidade; PAS: pressão arterial sistólica. ^**†**^ Utilizada a correlação de Pearson. ^**‡**^ Utilizada a análise de correlação parcial.*

### Resultados clínicos

**Figura 1:**
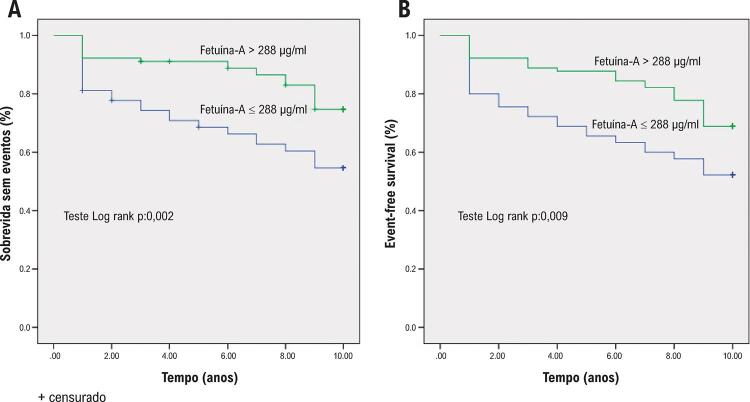
– Curvas de sobrevida de Kaplan-Meier de mortalidade por doença cardiovascular (A) e mortalidade global (B) de acordo com a fetuína-A.

Os resultados clínicos são apresentados na Tabela 3 . Durante um acompanhamento médio de 10 anos, foram registradas 71 mortes, das quais 62 foram devidas a DCV. Os índices de mortalidade global e por DCV foram significativamente mais altos no grupo com nível de fetuína-A mais baixo que no grupo com nível fetuína-A mais alto (44% versus 24%, p= 0,005; 48% versus 31%, p=0,022, respectivamente). As curvas de sobrevida acumulada demonstraram que os riscos de mortalidade global e por DCV aumentaram à medida que os níveis de fetuína-A diminuíram ( Figura 1 ).

**Tabela 3:** – Índices de mortalidade global e cardiovascular de acordo com os níveis de fetuína-A plasmática

No acompanhamento de 10 anos	Fetuína-A mais baixa (≤288 µg/ml) (n:90)	Fetuína-A mais alta (≤288 µg/ml) (n:90)	p-valor
Morte por DCV, n (%)	40 (44)	22 (24)	0,005
Mortalidade global, n, (%)	43 (48)	28 (31)	0,022

*DCV: doença cardiovascular.*

### Análise de regressão de Cox

As RC para mortalidade cardiovascular e global associadas ao nível de fetuína-A plasmática são apresentadas na Tabela 4 . Mesmo após a padronização para vários fatores confundidores (modelo 4), identificou-se que a fetuína-A é um preditor independente da mortalidade cardiovascular, como variável categórica e contínua (RC=0,46, IC 95%: 0,27-0,78 e RC=0,997, IC 95%: 0,994-0,999, respectivamente). Para a mortalidade global, após várias padronizações (modelo 4), identificou-se que a fetuína-A foi um preditor significativo como variável contínua (RC=0,997, IC 95%: 0,995-1,000). Ao mesmo tempo, houve uma tendência para a significância estatística da fetuína-A como variável categórica (p=0,085).

**Tabela 4:** – Razões de risco baseadas nos modelos de regressão de Cox para estimar os efeitos dos níveis de fetuína-A na mortalidade global e por doença cardiovascular

	Mortalidade por DCV		Mortalidade global	
	Razão de risco (IC 95%)	p-valor	Razão de risco (IC 95%)	p-valor
**Fetuína-A como variável categórica**				
Modelo 1	0,46 (0.27-0.78)	0,004	0,54 (0.34-0.88)	0,014
Modelo 2	0,46 (0.27-0.79)	0,005	0,55 (0.33-0.89)	0,016
Modelo 3	0,48 (0.28-0.83)	0,009	0,58 (0.35-0,95)	0,032
Modelo 4	0,55 (0.31-0.96)	0,036	0,64 (0.38-1.06)	0,085
**Fetuína-A como variável contínua**				
Modelo 1	0,995 (0.993-0.998)	0,001	0,996 (0.993-0.998)	0,001
Modelo 2	0,995 (0.993-0.998)	0,001	0,996 (0.993-0.998)	0,002
Modelo 3	0,996 (0.993-0.999)	0,003	0,996 (0.994-0.999)	0,005
Modelo 4	0,997 (0.994-0.999)	0,038	0,997 (0.995-1.000)	0,034

*Modelo 1: Não padronizado. Modelo 2: padronizado para idade e sexo. Modelo 3: padronizado além disso para tabagismo, histórico de diabetes mellitus, histórico de hipertensão, e nível de colesterol total. Modelo 4: padronizado além disso para nível de creatinina e fração de ejeção ventricular esquerda. IC: intervalo de confiança; DCV: doença cardiovascular.*

## Discussão

No presente estudo, demonstrou-se que os níveis mais altos de fetuína-A plasmática tinham risco mais baixo de mortalidade global e por DCV em pacientes com STEMI. Identificou-se que a associação era independente de fatores de risco estabelecidos para DCV, tais como, idade, tabagismo, hipertensão, diabetes mellitus, hiperlipidemia e creatinina. Além disso, identificou-se que o nível de fetuína-A plasmática estava inversamente correlacionado ao PCR-us, e diretamente correlacionado à glicemia de jejum.

Estudos prévios investigando a relação entre a fetuína-A e a mortalidade global e cardiovascular tiveram resultados conflitantes. Concentrações mais baixas de fetuína-A previram o aumento da mortalidade global e por DCV nas populações com doença renal terminal, pacientes de diálise, e população em geral.^7 , 12 , 13^ Lim et al.,^14^ estabeleceram que os índices de mortalidade de 6 meses eram significativamente mais altos em pacientes com STEMI com níveis baixos de fetuína-A em comparação aos pacientes com níveis altos de fetuína-A.^14^ Além disso, Chen et. al.,^15^ demonstraram que níveis de fetuína-A plasmática mais baixos estavam associados com um aumento do risco de mortalidade global e por DCV em pacientes com DAC estável, independentemente de fatores de risco de DCV tradicionais.^15^ Contrariamente, Roos et al.,^16^ demonstraram não existir associação significativa entre os níveis de fetuína-A sérica e eventos secundários de DCV em pacientes com DAC após o acompanhamento de 6 anos.^16^ Além disso, no estudo EPIC-Postdam, Weikert et al.,^17^ demonstraram que pacientes com concentrações altas de fetuína-A tinham risco 4 vezes mais alto de infarto do miocárdio e acidente vascular isquêmico em comparação aos sujeitos com níveis baixos de fetuína-A.^11^

As razões subjacentes para tais discrepâncias mencionadas acima não foram claramente identificadas, mas algumas explicações foram propostas. A calcificação vascular e a inflamação podem ter um papel importante no desenvolvimento da aterosclerose e DCV. A fetuína-A pode ajudar a prevenir o desenvolvimento de doenças cardiovasculares por suas funções anti-inflamatórias e anticalcificação.^17^ A fetuína-A tem um papel crucial no caminho anti-inflamatório após uma isquemia miocárdica, provavelmente facilitando o início do processo de cura.^4^ Entretanto, a fetuína-A está envolvida na patogênese do diabetes mellitus tipo 2 ao inibir a fosforilação do receptor de insulina tirosina, o que leva à resistência a insulina.^3^ Uma meta-análise recente confirmou a associação, demonstrando que um incremento DP do nível de fetuína-A resulta em um risco 23% mais alto de diabetes mellitus tipo 2 incidente.^18^ Além disso, a fetuína-A poderia levar à ativação do receptor do tipo Toll 4 e à migração do macrófago, resultando em inflamação do tecido adiposo e disfunção do adipócito.^19^ Com base nesses fatos, o nível alto de fetuína-A, e não o nível baixo de fetuína-A, pode ser associado a aterosclerose e DCV. Esse efeito duplo da fetuína-A pode ter causado resultados heterogêneos em status e condições diferentes. Sugere-se que, nos estágios iniciais de DCV, a fetuína-A exacerba essa doença devido a seus efeitos de promoção da resistência à insulina e dislipidemia; entretanto, em estágios posteriores de DCV, altas concentrações de fetuína-A tiveram resultados positivos devido a seu efeito anti-inflamatório e capacidade de evitar o depósito de cálcio vascular.^20 , 21^ Outra explicação possível para esses resultados contraditórios é o fato de que a fetuína-A é uma proteína de fase aguda, que pode ser reduzida marcadamente no stress agudo.^22^ Embora essa condição reflita o equilíbrio da inflamação aguda em síndromes coronárias agudas, ela pode ter efeitos ilusórios em resultados de longo prazo. Por fim, nesses estudos, os níveis de fetuína-A foram medidas utilizando-se vários imunoensaios com sensibilidades e especificidades diferentes.

No estudo atual, identificou-se que a alta concentração de fetuína-A estava associada a um resultado favorável após o STEMI. Os efeitos inibidores da fetuína-A em processos inflamatórios podem explicar essa observação. A inflamação tem um papel crucial na patogênese da aterosclerose e da SCA. Após o SCA, a inflamação se espalha por todo o miocárdio e está envolvida na cicatrização e na recuperação das funções miocárdicas.^23^ Entretanto, o papel de mediadores anti-inflamatórios em contra-atacar e modular o processo inflamatório parece ser crítico para evitar a cicatrização inadequada.^9^ Nos casos em que a inflamação não pode ser suficientemente limitada por citocinas anti-inflamatórias, a inflamação pode levar a consequências desfavoráveis, tais como fibrose cardíaca, dilatação crônica, insuficiência cardíaca e arritmia.^24^ Há muitas evidências que demonstram os efeitos prejudiciais de mediadores pró-inflamatórios na SCA.^4 , 25^ Dados recentes também sugerem que marcadores anti-inflamatórios podem ter valor similar.^26 , 27^ Feistritzer et al.,^28^ demonstraram que a fetuína-A baixa, associada a efeitos adversos de dimensões de enfartamento, função do ventrículo esquerdo, e remodelação após STEMI agudo.^28^ A baixa concentração de fetuína-A facilitará o processo inflamatório existente^29^ e a superprodução de citocinas cardiotóxicas, tais como o fator de necrose tumoral,^30 , 31^ que exporá os pacientes a um risco aumentado de remodelação de VE e recorrência de SCA. Além disso, a baixa concentração de fetuína-A pode ter um efeito deletério direto na função miocárdica. Merx et al.,^32^ relataram recentemente o avanço da fibrose cardíaca, calcificação, função diastólica notadamente prejudicada, e tolerância a isquemia, bem como resistência a catecolamina nos corações de ratos knockout com fetuína-A.^32^

No cenário de STEMI, o efeito anti-inflamatório da fetuína-A pode ser mais dominante para se determinar o prognóstico que seus outros efeitos. Isso pode explicar a relação entre os níveis altos de fetuína-A e baixa mortalidade após o STEMI. Devido à associação bifásica da fetuína-A com doença cardiovascular, dependendo do estágio da aterosclerose, os valores de fetuína-A podem estar restritos a pacientes com STEMI para avaliar esse prognóstico.

### Limitações do estudo

O presente estudo tem várias limitações a serem mencionadas. A limitação mais importante do presente estudo é que ele reflete uma experiência em um centro único com um número limitado de pacientes. Devido ao pequeno número de pacientes, o valor prognóstico da fetuína-A deve ser interpretado com cuidado nessa população. Nossos resultados são baseados em medições de fetuína-A de amostras de sangue únicas na admissão hospitalar. Os níveis de fetuína-A não foram avaliados após a fase aguda do infarto do miocárdio. A análise seria mais valiosa se a alteração da fetuína-A ao longo do tempo pudesse ser demonstrada com várias medidas. Por fim, este estudo não pôde considerar completamente todos os possíveis fatores confundidores associados aos níveis de fetuína-A plasmática ou desenvolvimento de DCV.

## Conclusão

O presente estudo demonstra que a baixa concentração de fetuína-A está associada a um prognóstico ruim para casos de STEMI no longo prazo. O impacto deletério de um processo inflamatório parece persistir após 10 anos de acompanhamento. Nossos achados fortaleceram estudos prévios demonstrando consistentemente o papel determinante dos mediadores anti-inflamatórios em síndromes coronárias agudas. São necessários estudos randomizados de larga escala posteriores para explicar a utilidade clínica dos níveis de fetuína-A na previsão do prognóstico pós-STEMI no acompanhamento.
