# Supplementary material for: Níveis mais Baixos de Fetuína-A Sérica estão Associados a um Maior Risco de Mortalidade em Dez Anos em Pacientes com Infarto do Miocárdio por Supradesnivelamento do Segmento ST
Source: Arq Bras Cardiol. 2021 Nov 17;118(1):14–21. [Article in Portuguese] doi: 10.36660/abc.20201057 (PMC8959055; doi:10.36660/abc.20201057)
Supplement: Translation

# Translation: Lower Serum Fetuin-A Levels are Associated with a Higher Ten-Year Mortality Risk in Patients with ST-Elevation Myocardial Infarction

**Authors:** Hakan Çakır, Selçuk Kanat, Hilal Çakır, Erhan Tenekecioğlu

**Affiliations:** 1 Turkey Kartal Kosuyolu Training and Research Hospital, Istanbul – Turkey; 2 Turkey Bursa Yuksek Ihtisas Training and Research Hospital, Bursa – Turkey; 3 Turkey Pendik State Hospital, Istanbul – Turkey

**Keywords:** ST Elevation Myocardial Infarction, Coronary Artery Disease, Mortality, Fetuin Alpha, Globulins, Anti- Inflammatory Agents, Echocardiography/methods

## Abstract

**Background:**

Fetuin-A is an anti-inflammatory and anti-calcification factor involved in the course of coronary artery disease (CAD). In line with these functions, fetuin-A has been investigated as a cardiovascular risk marker in many studies. However, the association between fetuin-A and the prognosis of CAD patients is still controversial.

**Objectives:**

The present study was conducted to identify the association between serum fetuin-A level and long-term cardiovascular disease (CVD) and all-cause mortality of ST-elevation acute myocardial infarction (STEMI).

**Methods:**

One hundred eigthy consecutive patients with STEMI were enrolled in the study. The study population was divided into subgroups (lower, ≤288 µg/ml; and higher, >288 µg/ml) according to the median fetuin-A level. Clinical follow-up data was obtained by annual contact with the patients or family members by telephone. The causes of death were also confirmed by the national health database. Two-sided p-values<0.05 were considered statistically significant.

**Results:**

During a median follow-up of 10 years, 71 deaths were recorded , 62 of whom died from CVD. Both CVD and all-cause mortality were found to be significantly higher in the lower fetuin-A group than the higher fetuin-A group (44% vs 24%, p= 0.005; 48% vs 31%, p= 0.022, respectively). In Cox regression proportional hazard analyses, fetuin-A was found to be an independent predictor of CVD and all-cause mortality.

**Conclusions:**

Low fetuin-A concentration is associated with a poor long-term prognosis after STEMI, regardless of the traditional cardiovascular risk factors. Our findings have strengthened previous studies that consistently demonstrate the determining role of anti-inflammatory mediators in acute coronary syndromes.

## Introduction

Fetuin-A, α2-Heremans-Schmid glycoprotein, is an abundant serum protein that is exclusively produced by the liver, tongue, placenta and adipose tissue.^1 , 2^ It plays a crucial role as a physiological inhibitor of insulin receptor tyrosine kinase, associated with insulin resistance and a negative acute phase reactant. It also regulates bone remodeling and calcium metabolism, and is an important inhibitor of calcium salt precipitation and vascular calcifications.^3^

Inflammation is a key process in atherosclerosis. Substantial evidence exists for the detrimental effect of pro-inflammatory cytokines on the cardiovascular system.^4^ During inflammation, proinflammatory cytokines, such as IL-1ß and IL-6, decrease the synthesis of fetuin-A in the liver. Decreased circulating fetuin-A levels constrains the activities of numerious anti-inflammatory mediators, thereby aggravating the inflammatory response. Fetuin-A is one of the inhibitors of calcification in soft tissue and vascular tree.^5^ Coronary artery calcification is regarded as an index of the severity of atherosclerotic vascular disease and may also predict future adverse cardiovascular events.

In line with these functions, fetuin-A has been investigated as a cardiovascular risk marker in many studies. Studies in end-stage renal disease (ESRD) populations have consistently shown that lower fetuin-A levels are associated with CVD-events and all-cause mortality.^6 - 8^ However, the relation between serum fetuin-A levels and the prognosis of coronary artery disease (CAD) patients is still controversial. High serum fetuin-A level is associated with a lower 1-year mortality in acute coronary syndromes (ACS).^9^ On the other hand, Parker et al.^10^ demonstrated that no significant association of fetuin-A with mortality or a CVD event was observed in patients with stable CAD.^10^ Weikert et al.^11^ reported a relationship between increased plasma fetuin-A levels and an higher risk of myocardial infarction (MI) and ischemic stroke in a large population-based cohort.^11^

The present study aimed to identify the association between the serum fetuin-A level and the long-term CVD and all-cause mortality of ST-elevation acute myocardial infarction (STEMI). We hypothesized that lower fetuin-A levels would be a prognosic marker for long-term mortality after STEMI, regardless of other CVD risk factors.

## Methods

### Study population

Between May 2009 and September 2009, all patients (n: 195) diagnosed with STEMI, and admitted to the hospital within 12 h of the onset of symptoms, were first enrolled in the study. Patients with cardiogenic shock within 24h were also included. STEMI was diagnosed in the presence of the two following criteria: persistent angina for ≥ 20 minutes and ST-segment elevation of ≥ 1 mm in ≥ 2 contiguous leads other than V2 to V3, or the presence of new left-bundle branch block. In leads V2 to V3, 2 mm of ST-segment elevation in men and 1.5 mm of ST-segment elevation in women was required for the STEMI diagnosis. Patients were managed according to guidelines; treatment was not affected by participation in the registry. Primary percutaneous coronary intervention (PCI) was preferred as the reperfusion technique for the entire study population. Patients were excluded if any of the following were present: culprit or critical lesion in the left main coronary artery, previous coronary artery bypass surgery, end-stage renal disease (creatinine clearance, <15 mL/min), active infection, chronic inflammatory disease, and known malignancy. Finally, the study population consisted of 180 patients. A complete physical examination was performed, and a detailed medical history was obtained from each case. Height, weight, and blood pressure (BP) were measured by trained nurses using standard protocols and procedures. Hypertension was defined as a systolic BP of ≥140 mmHg, a diastolic BP of ≥90 mmHg, or current treatment by any antihypertensive drug. Body mass index (BMI) was calculated as weight (kg) / height^2^(m^2^). Informed consent was obtained from all individual participants. The study was reviewed and approved by the institutional ethics committees.

### Biochemical measurement

Venous blood samples were collected from participants after overnight fasting. Serum was immediately separated from the cells by centrifugation at 3000g for 10 minutes, stored on ice at -70ºC until analyzed. Serum fetuin-A was measured using a commercially available human fetuin-A enzyme-linked immunosorbent assay kit (BioVendor Laboratory Medicine, Inc., Brno, Czech Republic). The intra- and inter-assay coefficients of variation for fetuin-A were less then 8%. High sensitive CRP (hs-CRP) levels were measured by immunonephelometric method (Image Immunochemistry System; Beckman Coulter, Inc., Fullerton, CA). Other biochemical parameters were measured using commercially available methods and kits.

### Echocardiography

A two-dimensional echocardiogram was performed in-hospital before discharge for the evaluation of left ventricle ejection fraction (LVEF), using modified the Simpson’s technique. The analysis was performed by two observers blinded to the clinical and angiographical data.

### Prospective follow-up and outcomes

Clinical follow-up data was obtained by annual contact with the patients or their family members by telephone after PCI. The causes of deaths were also confirmed by the national health database. Follow-up was completed in all patients. The primary outcomes of the study were cardiovascular mortality (ICD codes I00-I99) and all-cause mortality, which included cardiovascular mortality and death due to non-cardiac causes. The patients were screened until the end of February 2019 or the patient’s death.

### Statistical analysis

Continuous variables with normal distribution are expressed as mean ± standard deviation (SD) and continuous variables without normal distribution are expressed as median and interquartile range (IQR). Categorical data are expressed as absolute values and percentages. The Kolmogorov-Smirnov test was used to test the normal distribution. Differences in continuous variables between the groups were determined by independent sample t-test or the Mann-Whitney U-test, for variables with or without normal distribution, respectively. Categorical variables and proportions were analyzed by the chi-square test. The correlation between plasma fetuin-A and CVD risk factors was analyzed using the Pearson correlation. Survival curves for plasma fetuin-A subgroups were constructed by Kaplan-Meier method, differences in survival were assessed using the log-rank test.

The Cox proportional hazards model analysis was used to evaluate the association between serum fetuin-A and CVD and all-cause mortality in a 10-year follow-up. The first model was unadjusted. Model 2 was adjusted for age and sex. Based on model 2, model 3 was further adjusted for traditional cardiovascular risk factors, such as smoking, history of diabetes mellitus, history of hypertension, and total cholestrol level. Based on model 3, model 4 was further adjusted for creatinine level and LVEF. Fetuin-A was analyzed separately as both categorical and continuous variables. Results of Cox regression models were presented as hazard ratios (HR) and 95% confidence intervals (CI).

Two-sided p values <0.05 were considered statistically significant. All statistical analyses were performed with SPSS 20.0 (IBM SPSS Inc., Chicago, IL).

## Results

### Clinical and biochemical characteristics

The study population consisted of 180 patients with STEMI and were divided into lower- and higher-fetuin-A subgroups according to the median fetuin-A level upon admission. The demographic and biochemical characteristics of the baseline fetuin-A levels according to the subgroups (lower, ≤288 µg/ml; and higher, >288 µg/ml) are summarized in Table 1 . Patients from the lower fetuin-A group were older and had a higher prevalence of hypertension and greater serum creatinine and hs-CRP levels compared to the higher fetuin-A group. In the echocardiography, LVEF values were lower in the lower fetuin-A group.

**Table 1:** – Baseline Characteristics of the Study Population Stratified by the Median of Admission Fetuin-A Values (≤288 µg/ml vs >288 µg/ml)

Variables	Groups of Fetuin-A		p value
	Lower Fetuin-A (≤288 µg/ml) (n:90)	Higher Fetuin-A (>288 µg/ml) (n:90)	
Age,y	60.5±9.9	57.1±9.6	0.019
Sex, female/male	12/78	17/73	0.311
BMI, kg/m^2^	26.0±2.0	25.8±2.0	0.528
Cigarette smoking, n (%)	59 (65.6)	51 (56.7)	0.221
Hypertension, n (%)	32 (35.6)	19 (21.1)	0.032
Diabetes mellitus, n (%)	25 (27.8)	18 (20.0)	0.221
Total cholesterol, mg/dl	181±34	182±34	0.922
*Triglycerides, mg/dl	141 [102-194]	137 [103-181]	0.595
HDL cholesterol, mg/dl	33±6	35±6	0.059
LDL cholesterol, mg/dl	115±30	113±31	0.658
Hemoglobin, g/dl	12.7±1.46	13.0±1.18	0.240
Creatinin, mg/dl	1.10±0.74	0.84±0.30	0.002
Hs-CRP, mg/dl	1.91±2.31	0.86±1.56	<0.001
Peak troponin I, ng/ml	45.0±37.6	35.1±34.1	0.066
Prior MI, n (%)	6 (6.6)	4 (5.5)	0.756
Anterior wall MI, n (%)	35 (38.9)	28 (31.1)	0.274
İnferior or right ventricle MI, n (%)	40 (44.4)	54 (60.0)	0.037
Posterolateral wall MI, n (%)	15 (16.7)	8 (8.9)	0.118
Time to reperfusion, h	5.7±6.8	6.4±10.0	0.547
Tirofiban administration, n (%)	55 (61.1)	50 (55.6)	0.450
LVEF, %	42±7	45±6	0.016

Data are expressed as mean ± SD, n (%) or *median [IQR]. BMI: body mass index; HDL: high-density lipoprotein; Hs-CRP: high sensitivity C-reactive protein; MI: myocardial infarction; LDL: low-density lipoprotein; LVEF: left ventricular ejection fraction.

### Fetuin-A and CVD risk factors

The bivariate correlation analysis between serum fetuin-A and CVD risk factors is shown in Table 2 . The serum fetuin-A level was inversely correlated with hs-CRP and positively correlated with fasting glucose after adjustment for sex, age, and BMI.

**Table 2:** – Association of Plasma Fetuin-A with Cardiovascular Disease Risk Factors

	Correlation With Plasma Fetuin-A Levels			
	No Adjustment		Adjusted for Sex, Age, and BMI	
Variables	r	p-value^†^	R	p-value^‡^
Age, y	-0.245	0.001	-	-
BMI, kg/m^2^	0.031	0.681	-	-
SBP, mm Hg	0.084	0.263	0.038	0.616
DBP, mm Hg	0.063	0.403	0.018	0.810
Fasting glucose, mg/dl	0.288	<0.001	0.272	<0.001
Total cholesterol, mg/dl	0.056	0.456	-0.004	0.961
Triglycerides, mg/dl	0.083	0.268	0.073	0.333
HDL cholesterol, mg/dl	0.175	0.019	0.124	0.099
LDL cholesterol, mg/dl	-0.009	0.903	-0.078	0.302
Hs-CRP, mg/dl	-0.276	<0.001	-0.233	0.002

*BMI: body mass index; DBP: diastolic blood pressure; HDL: high-density lipoprotein; Hs-CRP: high sensitive C-reactive protein; LDL: low-density lipoprotein; SBP: systolic blood pressure.^**†**^ Pearson correlation analysis was used.^**‡**^ Partial correlation analysis was used.*

### Clinical outcomes

Clinical outcomes are shown in Table 3 . During a median follow-up of 10 years, 71 deaths were recorded, 62 of whom died of CVD. Both CVD and all-cause mortality were significantly higher in patients in the lower fetuin-A group than in the higher fetuin-A group (44% vs 24%, p= 0.005; 48% vs 31%, p=0.022, respectively). The Kaplan-Meier cumulative survival curves showed that the risks for all-cause and CVD mortality increased as the fetuin-A levels declined ( Figure 1 ).

**Table 3:** – Cardiovascular and All-Cause Mortality Rates According to the Plasma Fetuin-A Levels

At 10-year follow-up	Lower Fetuin-A (≤288 µg/ml) (n: 90)	Higher Fetuin-A (>288 µg/ml) (n: 90)	p-value
CVD death, n (%)	40 (44)	22 (24)	0.005
All-cause death, n, (%)	43 (48)	28 (31)	0.022

*CVD: cardiovascular disease.*

**Figure 1:** – Kaplan-Meier survival curves of cardiovascular disease (A) and all-cause mortality (B) according to the Fetuin-A.

### Cox regression analysis

The HRs for all-cause and cardiovascular mortality associated with the plasma fetuin-A level are presented in Table 4 . Even after adjustment for multiple confounding factors (model 4), fetuin-A was found to be an independent predictor of cardiovascular mortality, both as categorical and continuous variables (HR=0.46, 95% CI: 0.27-0.78 and HR=0.997, 95% CI: 0.994-0.999, respectively). For all-cause mortality, after multiple adjustments (model 4), fetuin-A was found to be a significant predictor as a continuous variable (HR=0.997, 95% CI: 0.995-1.000). Meanwhile, there was a trend toward statistical significance for fetuin-A as a categorical variable (p=0.085).

**Table 4:** – Hazard Ratios based on Cox Regression Models to Estimate the Effects of Fetuin-A Levels on Cardiovascular Disease and All-Cause Mortality

	CVD Mortality		All-cause Mortality	
	Hazard Ratio (95% CI)	p-value	Hazard Ratio (95% CI)	p-value
**Fetuin-A as a categorical variable**				
Model 1	0.46 (0.27-0.78)	0.004	0.54 (0.34-0.88)	0.014
Model 2	0.46 (0.27-0.79)	0.005	0.55 (0.33-0.89)	0.016
Model 3	0.48 (0.28-0.83)	0.009	0.58 (0.35-0,95)	0.032
Model 4	0.55 (0.31-0.96)	0.036	0.64 (0.38-1.06)	0.085
**Fetuin-A as a continuous variable**				
Model 1	0.995 (0.993-0.998)	0.001	0.996 (0.993-0.998)	0.001
Model 2	0.995 (0.993-0.998)	0.001	0.996 (0.993-0.998)	0.002
Model 3	0.996 (0.993-0.999)	0.003	0.996 (0.994-0.999)	0.005
Model 4	0.997 (0.994-0.999)	0.038	0.997 (0.995-1.000)	0.034

*Model 1: Unadjusted. Model 2: adjusted for age and sex. Model 3: further adjusted for smoking, history of diabetes mellitus, history of hypertension and total cholestrol level. Model 4: further adjusted for creatinin level and left ventricular ejection fraction. CI: confidence interval; CVD: cardiovascular disease.*

## Discussion

The present study demonstrated that higher plasma fetuin-A levels had a lower risk of CVD and all-cause mortality in patients with STEMI. This association was found regardless of established risk factors for CVD, such as age, smoking, hypertension, diabetes mellitus, hyperlipidemia and creatinine. Moreover, the plasma fetuin-A level was found to be inversely correlated with hs-CRP and positively correlated with fasting glucose.

Previous studies investigating the relationship between fetuin-A and cardiovascular and all-cause mortality had conflicting results. Lower fetuin-A concentrations have predicted increased CVD and all-cause mortality in end-stage renal disease populations, dialysis patients and general population.^7 , 12 , 13^ Lim et al.^14^ established that 6-month mortality rates were significantly higher in STEMI patients with low fetuin-A levels compared to the patients with high fetuin-A levels.^14^ Chen et al.^15^ also showed that lower plasma fetuin-A levels were associated with an increased risk of all-cause and CVD mortality in patients with stable CAD regardless of traditional CVD risk factors.^15^ By contrast, Roos et al.^16^ demonstrated no significant association between serum fetuin-A levels and secondary CVD events in patients with CAD after a 6-year follow up.^16^ Moreover, in the EPIC-Potsdam study, Weikert et al.^17^ showed that patients with high fetuin-A concentrations had a 4-fold increased risk for myocardial infarction and ischemic stroke when compared to the subjects with low fetuin-A levels.^11^

The underlying reasons for such discrepancies mentioned above have not been clearly identified, but some explanations have been proposed. Inflammation and vascular calcification play an important role in the development of atherosclerosis and CVD. Fetuin-A might help to prevent the development of cardiovascular diseases through its anti-inflammatory and anti-calcification functions.^17^ Fetuin A plays a crucial role in the inflammatory pathway following myocardial ischemia, likely facilitating the initiation of the healing process.^4^ However, Fetuin-A is involved in the pathogenesis of type 2 diabetes mellitus by inhibiting insulin-receptor tyrosine phosphorylation, which leads to insulin resistance.^3^ A recent meta-analysis confirmed this association by showing that one SD increment of a fetuin-A level results in a 23% greater risk of incident type 2 diabetes mellitus.^18^ Fetuin-A may also lead to toll-like receptor-4 activation and macrophage migration, resulting in adipose tissue inflammation and adipocyte dysfunction.^19^ Based on these facts, a high fetuin-A level, rather than a low fetuin-A level, might be associated with atherosclerosis and CVD. This dual effect of fetuin-A may have caused heteregenous results in different statuses and conditions. It is suggested that, in early stages of CVD, fetuin-A exacerbates the disease due to its effects to promote insulin resistance and dyslipidemia; however, in later stages of CVD, high concentrations of fetuin-A have positive results due to its anti-inflammatory effect and ability to prevent vascular calcium deposition.^20 , 21^ Another possible explanation of these contradictory results is the fact that fetuin-A is an acute phase protein, which can be markedly reduced in acute stress.^22^ Although this condition reflects the acute inflammation balance in acute coronary syndromes, it may have misleading effects on long-term results. Lastly, in those studies, fetuin-A levels were measured using various immunoassays with different sensitivity and specificity.

In the current study, a high fetuin-A concentration was found to be associated with a favorable outcome after STEMI. The inhibitory effects of fetuin-A on inflammatory processes may explain this observation. Inflammation plays a crucial role in the pathogenesis of atherosclerosis and ACSs. Following ACS, inflammation spreads throughout the entire myocardium and is involved in the healing and restoration of myocardial functions.^23^ However, the role of anti-inflammatory mediators in counteracting and modulating the inflammatory process appears critical to avoid inadequate healing.^9^ In cases where the inflammation cannot be limited by anti-inflammatory cytokines sufficiently, inflammation may promote unfavorable consequences, such as cardiac fibrosis, chronic dilatation, heart failure, and arrhythmia.^24^ A wide range of evidence shows harmful effects of pro-inflammatory mediators in ACS.^4 , 25^ Recent data also suggest that anti-inflammatory markers may have similar value.^26 , 27^ Feistritzer et al.^28^ showed that low fetuin-A is associated with an adverse effect on infarct size, left ventricular function, and remodelling after acute STEMI.^28^ A low fetuin-A concentration will facilitate the ongoing inflammatory process^29^ as well as the overproduction of cardiotoxic cytokines, such as the tumor necrosis factor,^30 , 31^ which will expose patients to an increased risk of LV remodeling and recurrence of ACS. In addition, a low fetuin-A concentration may have a direct deleterious effect on myocardial function. Merx et al.^32^ recently reported the progress of cardiac fibrosis, calcification, notably impaired diastolic function, and tolerance to ischemia, as well as catecholamine resistance in the hearts of fetuin-A knockout mice.^32^

In the setting of STEMI, the anti-inflammatory effect of fetuin-A may be more dominant in determining the prognosis than its other effects. This may explain the relationship between high fetuin A levels with low mortality after STEMI. Due to the biphasic association of fetuin-A with vascular disease, depending on the stage of atherosclerosis, fetuin-A values might be restricted to STEMI patients to assess the prognosis.

### Study limitations

The present study has several limitations that should be mentioned. The most important limitation of the current study is that it reflects a single center experience with a limited number of patients. Because of the small number of patients, the prognostic value of fetuin-A should be interpreted with caution in this population. Our results are based on fetuin-A measurements from single blood samples upon admission. Fetuin-A levels were not evaluated after the acute phase of the myocardial infarction. The analysis would be more valuable if the change of fetuin-A over time could be shown with several measures. Lastly, our study could not completely consider all potential confounding factors associated with the plasma fetuin-A levels or CVD development.

## Conclusion

The current study shows that a low fetuin-A concentration is associated with a poor long-term prognosis in STEMI. The deleterious impact of an inflammatory process appears to persist after a 10-year follow-up. Our findings have strengthened previous studies, which consistently demonstrate the determining role of anti-inflammatory mediators in acute coronary syndromes. Further large-scale and randomized studies are needed to explain the clinical usefulness of fetuin-A levels in the prediction of prognosis after STEMI during follow-up.
